# Autonomous dysfunction in Lyme neuroborreliosis. A case report

**DOI:** 10.1002/ccr3.1494

**Published:** 2018-03-22

**Authors:** Bent‐Are Hansen, Torgeir Finjord, Øyvind Bruserud

**Affiliations:** ^1^ Department of Microbiology Førde Hospital Førde Norway; ^2^ Department of Medicine Førde Hospital Førde Norway; ^3^ Section for Endocrinology Department of Clinical Science University of Bergen Bergen Norway

**Keywords:** Autonomous dysfunction, constipation, Lyme neuroborreliosis, tick‐borne infection

## Abstract

The diagnosis of Lyme neuroborreliosis should be considered whenever a patient presents with neurological symptoms and comes from an endemic area. However, atypical clinical presentation occurs including gastrointestinal manifestations because of autonomous dysfunction.

## Introduction

Lyme borreliosis is a tick‐borne infection caused by *Borrelia burgdorferi* (*B. burgdorferi*) sensu lato. The most frequent clinical manifestations are erythema migrans and Lyme neuroborreliosis. Lyme neuroborreliosis can be further divided into an early and a late stage, and typically includes a wide range of clinical symptoms such as radicular pain, headache, fatigue, paresthesia, peripheral facial nerve palsy, and meningeal signs. However, autonomous dysfunction is also described in the context of Lyme neuroborreliosis. In the following case report, we present a 60‐year‐old male patient with severe gastrointestinal symptoms and a reversible autonomous dysfunction caused by acute Lyme neuroborreliosis.

## Case Report

A 62‐year‐old man presented with persistent epigastric pain at the emergency department. The pain had lasted for 4–5 weeks and it began with a rash in the right hypochondriac area. He described a constant pain with episodic worsening. There was no relief of pain with defecation or increase in pain due to food intake. During these weeks, several differential diagnoses were considered. The present symptoms were initially perceived as herpes zoster and treated with aciclovir and pregabalin. Despite treatment, the pain was increasing and he developed additional symptoms including nausea, lethargy, decreased appetite, constipation, decreased size and force of the urinary stream, and a 5–7 kg weight loss. Computed tomography (CT) revealed some small gallbladder stones. The symptoms were therefore interpreted as possible biliary colic but acute surgery was not recommended. His past medical history was significant for migraines, and periodically he had cognitive therapy for health anxiety. Four years ago, a colonoscopy revealed polyps and he used esomeprazole 40 mg on a daily basis because of reflux esophagitis. There were no diseases in his family.

The patient was hemodynamically and respiratory stable at admission with a blood pressure of 132/89 mmHg, heart rate was regular with 89 beats per minute, temperature was 36.9°C, saturation was 98%, and the respiratory rate was 10 per minute. However, his general condition was reduced. Physical examination revealed tenderness in the epigastrium by palpation. To notice, no specific neurological findings were present. Laboratory tests were all within the normal range and showed leukocyte count 9.7*10^9^/L (references: 4.3–10.7*10^9^/L), hemoglobin 15.2 g/dL (references: 13.4–17.0 g/dL), sedimentation rate 7 (references: <21), CRP < 1 mg/L (references: <5 mg/L), ALAT 29 U/L (references: <70 U/L), ALP 51 U/L (references: <105 U/L), GT 30 U/L (references: <115 U/L), amylase 61 U/L (references: <120 U/L), bilirubin 19 μmol/L (references: 5–25 μmol/L), and PSA 0.63 μg/L (references: <4 μg/L). Further examination with gastroscopy, colonoscopy, and a new CT abdomen was performed with normal findings. Based on the findings described above, no critical somatic diseases were found. In addition; the patient suggested that some part of his symptoms could be part of a functional state. He was therefore discharged home.

However, 1 week later, the patient was hospitalized because his general practitioner did not find it reasonable that his anxiety could explain his current symptoms. His clinical condition was unchanged. He lived in an endemic area for ticks before onset of symptoms, and even though he did not remember any recent tick bites, he reported several previous bites. Antibodies against *B. burgdorferi* were tested in Enzygnost Lyme link VlsE/IgG and Enzygnost Borreliosis IgM (DADE Behring, Marburg, Germany). Borrelia IgG was found strongly positive (>260 U/mL) (references: <6 U/mL) and Borrelia IgM was negative (77% of cut off value). Based on the high serological titers of *B. burgdorferi* IgG, previous tick bites, and a rash, a lumbar puncture was performed. Surprisingly, the cell count of the cerebrospinal fluid (CSF) showed leukocyte count 110*10^6^/L (no differential count was performed), protein 2.09 g/L (references: <0.60 g/L), and significant low glucose 2.8 mmol/L (references: 2/3 of serum glucose). Antibodies against *B. burgdorferi* in CSF were tested in an enzyme immunoassay (IDEIA Lyme Neuroborreliosis). *Borrelia burgdorferi* IgG index was 16.6 (references: <0.3) and *B. burgdorferi* IgM index was 10.4 (references: <0.3) which indicate intrathecal production of specific antibodies consistent with neuroborreliosis. He was then given intravenous ceftriaxone for 21 days according to the recommendation from the Norwegian Directorate of Health (https://helsedirektoratet.no/retningslinjer/antibiotika-i-sykehus). He has been under the surveillance in the outpatient clinic for the last 3 months. His weight is now normalized and he has no problems with constipation. The abdominal pain is almost gone. His main problem is fatigue but this is gradually improving.

## Discussion

Lyme disease is a tick‐borne disease caused by *B. burgdorferi* spirochetes. The disease occurs in stages and can affect multiple tissues and organs, making the clinical manifestations highly variable 1. However, a characteristic expanding skin lesion, termed erythema migrans, is typically the first symptom. This skin lesion resolves even without treatment, and only a minority of patients can recall tick bites or remember having cutaneous lesions. The pathogens can spread and afflict other skin regions, the heart, joints, or the nervous system 1. This process usually occurs within weeks or months, often following a period of latency 1. Neurological manifestations are reported in about 10% of patients with Lyme disease 2, most frequent symptoms being radicular pain, headache, paresthesia, peripheral facial nerve palsy, and meningeal signs. Laboratory evidence of infection is essential for the diagnosis, and the hallmark of acute infection is inflammatory CSF changes. Unfortunately, because of low sensitivities, direct detection methods for *B. burgdorferi* are of limited use in the diagnosis of Lyme neuroborreliosis and, therefore, indirect demonstration of infection using serology is the method of choice 3. Most patients experience full recovery after 2–3 weeks of antibiotics. Recently, a posttreatment Lyme disease syndrome is described because several patients report nonspecific symptoms such as fatigue following antibiotic treatment for Lyme neuroborreliosis 4.

Our patient in the case report presented atypical symptoms of Lyme neuroborreliosis. Despite no recent history of tick bites or typical neurological findings, the CSF findings were diagnostic for Lyme neuroborreliosis. To speculate, his initial rash and pain on the upper part of abdomen were possibly an erythema migrans and radiculoneuritis but were misdiagnosed as herpes zoster. However, autonomous dysfunction is reported as a rarely manifestation in the context of Lyme neuroborreliosis 5, and pseudoobstruction and constipation are described in a very few previous case reports 6, 7, 8. Moreover, the response to specific therapy for Lyme disease on the patients gastrointestinal symptoms suggests that the former was likely the result of a reversible autonomic neuropathy or dysfunction, which probably would develop to a fulminant pseudoobstruction if correct treatment was not initiated.

## Conclusion

The diagnosis of Lyme neuroborreliosis should be considered whenever a patient presents with neurological symptoms and comes from an endemic area. However, atypical clinical presentation occurs including gastrointestinal manifestations because of autonomous dysfunction.

## Conflict of Interest

None declared.

## Authorship

BH and ØB: Planned, wrote the manuscript, revised the manuscript, and did the literature search. TF: Involved in the medical care of the patient, planned, and revised the manuscript.
